# Commonalities and Differences in the Transcriptional Response of the Model Fungus *Saccharomyces cerevisiae* to Different Commercial Graphene Oxide Materials

**DOI:** 10.3389/fmicb.2020.01943

**Published:** 2020-08-11

**Authors:** Felix Laguna-Teno, Maria Suarez-Diez, Juan Antonio Tamayo-Ramos

**Affiliations:** ^1^International Research Centre in Critical Raw Materials-ICCRAM, University of Burgos, Burgos, Spain; ^2^Laboratory of Systems and Synthetic Biology, Wageningen University & Research, Wageningen, Netherlands

**Keywords:** *Saccharomyces cerevisiae*, biological response, commercial graphene oxide, chelating agent, RNA isolation, transcriptomics, differential expression

## Abstract

Graphene oxide has become a very appealing nanomaterial during the last years for many different applications, but its possible impact in different biological systems remains unclear. Here, an assessment to understand the toxicity of different commercial graphene oxide nanomaterials on the unicellular fungal model organism *Saccharomyces cerevisiae* was performed. For this task, an RNA purification protocol was optimized to avoid the high nucleic acid absorption capacity of graphene oxide. The developed protocol is based on a sorbitol gradient separation process for the isolation of adequate ribonucleic acid levels (in concentration and purity) from yeast cultures exposed to the carbon derived nanomaterial. To pinpoint potential toxicity mechanisms and pathways, the transcriptome of *S. cerevisiae* exposed to 160 mg L^–1^ of monolayer graphene oxide (GO) and graphene oxide nanocolloids (GOC) was studied and compared. Both graphene oxide products induced expression changes in a common group of genes (104), many of them related to iron homeostasis, starvation and stress response, amino acid metabolism and formate catabolism. Also, a high number of genes were only differentially expressed in either GO (236) or GOC (1077) exposures, indicating that different commercial products can induce specific changes in the physiological state of the fungus.

## Introduction

Graphene oxide is a nanomaterial of great industrial interest, and many public and private initiatives have been launched during the last decade for the development of new technologies around this 2D carbon derived product (Shapira et al., 2016). New applications based on graphene oxide are expected to increase the chance of its environmental release, which could lead to unsafe human and ecosystem exposure levels. Therefore, any possible risks associated to graphene oxide applications and release need to be well-understood (Fadeel et al., 2018), particularly considering its morphological and physical properties, which suggest a potential risk to the health of humans and the environment. In fact, attention is being drawn to the safety assessment of carbon derived nanomaterials in different biological systems, including graphene oxide, by the scientific community.

In most cases, graphene oxide risk assessment studies have been focused on mammalian cell lines and laboratory animals, where different mechanisms associated to its potential toxicity have been determined, namely, physical destruction, induction of oxidative stress, DNA damage, inflammatory response, apoptosis, autophagy, and necrosis (Sanchez et al., 2012; Ou et al., 2016; Ema et al., 2017). Although the biological impact of the nanomaterial has been also studied on microbial systems, only a limited number of studies have explored the toxicity mechanisms based on gene expression analysis (Chen et al., 2017; Yu et al., 2017; Zhu et al., 2017). Graphene has strong cytotoxicity toward bacteria (Liu et al., 2011), while little has been reported on its antifungal activity (Asadi Shahi et al., 2019). Most research works studying fungal interactions with graphene derivatives have focused on the nanomaterial functionalization with antifungal compounds, the development of cost-effective methods for surface modified graphene, or the integration of the cellular physiology with electrical read outs (Kempaiah et al., 2011; Khanra et al., 2012; Li et al., 2013; Valentini et al., 2016; Farzanegan et al., 2018). Still, the specific fungal responses to the presence of graphene oxide in the environment are poorly understood, as well as the possible physiological changes or the induction of specific toxicity pathways. Few research works have been done using the fungal genetic model *Saccharomyces cerevisiae*, to understand the potential toxicity of graphene oxide and other carbon derived nanomaterials (Bayat et al., 2014; Yu et al., 2017; Zhu et al., 2017, 2018), highlighting the need for more thorough studies assessing the global cellular response.

The yeast *S. cerevisiae* is one of the most widely used eukaryotic systems to understand basic molecular processes, therefore it is an ideal model to identify potential toxicity pathways induced by graphene oxide in fungi. Previous reports studying the toxicological effects of graphene oxide in this unicellular organism, show that an acute exposure leads to significant effects on cell viability and proliferation, due to mitochondria-mediated apoptosis, which could be associated with oxidative stress (Zhu et al., 2017). Also, at sublethal concentrations, cell growth and metabolism were reduced, possibly due to the iron chelating properties of graphene oxide (Yu et al., 2017). In this regard, a relevant binding capacity for metal ions and positively charged organic molecules has been assigned to this nanomaterial, through electrostatic interaction and coordination (Ali et al., 2019). This feature allows its use in the efficient removal of potentially toxic elements from contaminated aqueous media, but it could also impact nutrient bioavailability for the organisms present in a certain environment. Additionally, previous studies have reported that distinct graphene oxide products can have different reactivity against biological systems and biomolecules, possibly due to differences in their elemental composition or in morphological features (Antón-Millán et al., 2018; Li et al., 2018; Domi et al., 2019). Therefore, to assess whether different commercial graphene oxide products could induce different toxicity responses in *S. cerevisiae*, two graphene derivatives: monolayer graphene oxide (GO) and graphene oxide nanocolloids (GOC) were selected and the global transcriptional response of the yeast was compared. For this task, an optimized protocol for RNA isolation from fungal cells exposed to graphene oxide, was developed too.

## Materials and Methods

### Materials and Reagents

Most of the chemicals and reagents were purchased from Sigma-Aldrich and Thermo Fisher Scientific. The graphene derivatives were obtained from different suppliers as well: Graphene oxide nanocolloids (GOC; ref: 795534; lot: MKCD9594) were purchased from Sigma-Aldrich, and monolayer graphene oxide (GO; C309/GORB014/D1) was purchased from Graphenea. Working stock suspensions of both nanomaterial types were obtained using ultrapure water, at a final concentration of 1000 mg L^–1^, and were sonicated using a Branson Sonifier Cell Disruptor Model SLPe, for 5 min, using an amplitude of 40%.

#### *S. cerevisiae* Cells Exposure to Graphene Oxide Nanomaterials and RNA Isolation

*S. cerevisiae* cells were pre-grown on YPD medium in an orbital shaker (185 rpm, 30°C) until an O.D._600 nm_ = 1 was reached. Cells were harvested, washed with PBS and resuspended in 50 mL (O.D._600 nm_ = 1) of fresh YPD medium containing 160 mg L^–1^ of either GO or GOC, or without the presence of nanoparticles (negative control). Exposure cultures were performed in sterile 250 mL Erlenmeyer flasks, for 24 h (185 rpm, 30°C), growing two biological replicates per condition. Afterward, *S. cerevisiae* cells were harvested, resuspended with cold PBS and separated from the nanomaterials following the gradient centrifugation protocol described in the first paragraph of the Results and Discussion section, employing a Thermo ST 16R Sorvall centrifuge. All separation steps were performed at 4°C. Once yeast cells were separated from the graphene oxide nanoparticles, RNA isolation was performed using Thermo Fisher Scientific reagents, following the TRIzol^*TM*^ Plus RNA Purification Kit user guide (Pub. No. MAN0000561), with minor modifications. Briefly, yeast aliquots were pelleted by centrifugation (13,000 g, 4°C) and were subsequently resuspended in 1 mL of TRIzol^*TM*^ reagent and transferred to commercial 2 mL tubes prefilled with glass beads (Lysing Matrix C; MP). Yeast samples were disrupted using a FastPrep-24 Instrument (MP). After disruption, 200 μL of chloroform were added and the mix was homogenated for 10 s. The mix was poured into Phasemaker tubes (2 mL) and centrifuged at 13,000 g in a table-top centrifuge. The RNA present in the water phase was purified using the PureLink^*TM*^ RNA Mini Kit (Thermo), following the manufacturer’s instructions.

### RNA Quality Control and Sequencing

RNA integrity was assessed with an Agilent 2100 system, and only high quality samples (RIN value ≥ 8) were selected for whole transcriptome shotgun sequencing. Total RNA was sent for whole transcriptome sequencing to Novogene Bioinformatics Technology Co. Ltd. (HongKong, China). After mRNA purification (starting with 1 μg of total RNA per sample), sequencing libraries were generated using NEB Next^®^ Ultra^*TM*^ RNA Library Prep Kit for Illumina^®^ (NEB, United States) following manufacturer’s recommendations and index codes were added to attribute sequences to each sample. First, mRNA purification was done using poly-T oligo-attached magnetic beads, and fragmentation was carried out using divalent cations under elevated temperature in NEB Next First Strand Synthesis Reaction Buffer (5X). Subsequently, first strand cDNA was synthesized with random hexamer primer and M-MuLV Reverse Transcriptase (RNase H-) and second strand cDNA synthesis was done with DNA Polymerase I and RNase H. Remaining overhangs were converted into blunt ends via exonuclease/polymerase activities. After adenylation of 3’ ends of DNA fragments, NEBNext Adaptor with hairpin loop structure were ligated to prepare for hybridization. To select cDNA fragments of preferentially 150–200 bp in length, the library fragments were purified with AMPure XP system (Beckman Coulter, Beverly, United States). Then 3 μl USER Enzyme (NEB, United States) was used with size-selected, adaptor-ligated cDNA at 37°C for 15 min followed by 5 min at 95°C before PCR. Subsequently, PCR was performed with Phusion High-Fidelity DNA polymerase, Universal PCR primers and Index (X) Primer. Finally, PCR products were purified (AMPure XP system) and library quality was assessed on the Agilent Bioanalyzer 2100 system. After sample preparation, the clustering of the index-coded samples was performed on a cBot Cluster Generation System using PE Cluster Kit cBot-HS (Illumina) according to the manufacturer’s instructions, followed by the library preparations sequencing on an Ilumina Hiseq4000 and 125 bp/150 bp paired-end reads were generated. Afterward, the original raw data from Illumina was transformed to Sequenced Reads by base calling and data quality control was done with the Casava v1.8 software.

### RNA-Seq Data Processing and Analysis

Reads were pre-processed using FastqPuri for quality control and adapter, contamination and quality filtering (Pérez-Rubio et al., 2019). Reads with adapter contamination were removed, as well as the ones with 50% of the bases with quality below 20. Also, reads with a percentage of unidentified bases greater than 10% were also removed. Latest assembly of the reference genome for this strain was retrieved from Ensembl, revision 97, genome accession number (GCA_000146045.2). Reads were mapped to the genome using Star v2.7.2b (Dobin et al., 2013). The genome was indexed specifying the read length to improve accuracy. The mapping was done using two pass method. Number of reads for each genome feature were retrieved using featureCounts (Liao et al., 2014). Total number of reads are summarized in the Supplementary Table 1. Data have been submitted to the European Nucleotide Archive and can be found under accession number PRJEB34525. Read counts per gene were normalized and differential expression was computed using DESeq2 v 1.24, with default parameters except for the alpha threshold that was set to 0.05 (Love et al., 2014). Variance stabilizing transformation considering the experimental design was performed using the “rlog” command prior to principal component analysis. Enrichment analysis for selected groups of genes were performed using the hypergeometric function to model the background probability and the Benjamini–Hochberg procedure was used to control the false discovery rate (FDR) and correct for multiple testing. Gene ontology enrichment was performed using clusterProfiler v3.12.0, topGO and DOSE (Yu et al., 2012). Annotation files, both GAF and OBO were downloaded from Gene Ontology, release “2019-04-17” and pathway information was retrieved from KEGG (Kanehisa, 2000). The version 3.6.0 of R was used to perform the statistical analysis and visualizations were done with ggplot2 v3.2.1 (Wickham, 2009). Additional information about each gene was obtained from The Saccharomyces Genome Database (SGD) (Cherry et al., 2012).

## Results and Discussion

### Optimization of RNA Isolation From *S. cerevisiae* Cells Exposed to Different Commercial Graphene Oxide Products

**FIGURE 1:**
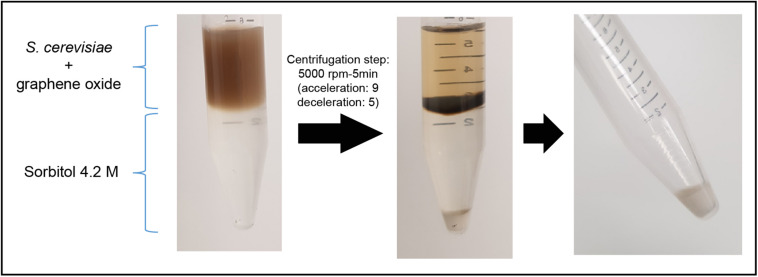
*S. cerevisiae* cells separation from graphene oxide through density gradient centrifugation.

The ability of graphene oxide to adsorb single-stranded nucleic acids (Park et al., 2013) is a burden for the isolation of RNA from cells that have been exposed to the nanomaterial. In the presence of this nanomaterial, the obtention of high-quality total RNA from *S. cerevisiae* cells, in enough amounts to be used for RNAseq analysis, can only be achieved if a nanoparticles-cells separation step is introduced prior to the start of the RNA isolation protocol. In fact, we failed in isolating total RNA from *S. cerevisiae* cells (strain BY4741) after an exposure experiment to GO and GOC, when they were not previously separated from the nanomaterials. Zhu and collaborators used a density gradient centrifugation protocol (Zhu et al., 2016), to separate graphene oxide nanoparticles from yeast cells for RNA purification (Zhu et al., 2017). However, considering the high mRNA turnover of some genes, we considered that the reported separation protocol used a too long centrifugation step (30 min) that might affect RNA integrity. Therefore, we decided to optimize the graphene oxide-cells separation protocol by speeding up the process, modifying the gradient centrifugation protocol, using a Thermo ST 16R Sorvall centrifuge, managing to efficiently separate the yeast cells from GO and GOC, by employing the following steps: once the cells exposure to the selected nanomaterials was finished, cells were harvested by centrifugation (5000 rpm, 4°C; acceleration: 9, deceleration: 9) and resuspended in cold PBS (2.5 mL). The resuspended cells were carefully overlayed in a concentrated sorbitol solution (4.2 M; 3 mL) prepared in PBS too and contained in disposable 15 mL tubes previously stored on ice. Subsequently, a gradient centrifugation was performed (5000 rpm, 4°C; acceleration: 9, deceleration: 5). As displayed in Figure 1, the separation between yeast cells and the graphene oxide nanoparticles was possible with the described optimized protocol, and the isolation of high quality total RNA for transcriptomics analysis was successful.

### Transcriptional Response of *S. cerevisiae* Cells to Different Graphene Oxide Products

To assess the impact of commercial graphene oxide products, GO and GOC, on *S. cerevisiae* cells, a comparative transcriptomics analysis was done. In a previous study, we characterized both nanomaterials and observed differences in their composition and their oxidative stress inducing capacity in yeast, although it was challenging to associate their toxicological potential to their physico-chemical characteristics due to the many different variables that could be involved, such as lateral dimension, surface structure, functional groups, purity and protein corona (Domi et al., 2019). GO and GOC showed a wide lateral size distribution (from the nanometric to the micrometric scale), with a flake thickness of 1–2 nm. Their chemical composition analysis, performed through ATR-FTIR, ICP-MS, and XPS, revealed both nanomaterials were similar in oxygen functional groups content, while significant differences in the concentration of metals, metalloids and non-metal elements were observed between both nanomaterials. The content of metallic and metalloid elements in both nanomaterials was low, but higher in GOC, while S species were more abundant in GO. The presence of organosulfate groups in graphene oxide is responsible for part of the reactivity of this nanomaterial type, such as in the immobilization of adsorbed species (Eigler et al., 2013). However, we could not get insights on the type of S species (e.g., organic or inorganic) present in both graphene oxide products. Overall, any of the differences observed between both nanomaterial types, as well as other non-identified factors, could be responsible for the distinct toxicological response displayed by the cellular systems used as toxicity models at viability, vitality and oxidative stress levels. In case of *S. cerevisiae*, GO showed a higher capacity than GOC to induce oxidative stress, while differences observed in viability after the exposure to both nanomaterials were not significant (Domi et al., 2019). The study of the global transcriptional response of *S. cerevisiae* cells to the presence of each nanoproduct could provide additional insights into the common and/or product-specific molecular mechanisms behind their toxicity inducing factors. With this purpose, we decided to expose yeast cells to GO and GOC for 24 h and to study their global transcriptional signature. Concentrations of GO and GOC (160 mg L^–1^) were selected based on ranges used in similar studies assessing the toxicological impact of graphene derivatives in different organisms including fungi (Domi et al., 2019; Suarez-Diez et al., 2020), and specifically the works of Yu et al. (2017) and Zhu et al. (2017), that analyze the impact of graphene oxides, similar to the ones here studied. Yeast cells total RNA was isolated as described in the previous section and it was analyzed using the Illumina sequencing system (further details can be found in the “Materials and Methods” section). The obtained reads were mapped to the *S. cerevisiae* BY4741 genome. Supplementary Table 1 provides summarizing information on this process. The reads that could be uniquely mapped to the *S. cerevisiae* genome ranged between 89.3 and 92.4%, and 84.1–86.1% of the reads mapped to exonic regions in the genome. These numbers indicate the high quality of the RNA generated using our optimized protocol.

**FIGURE 2:**
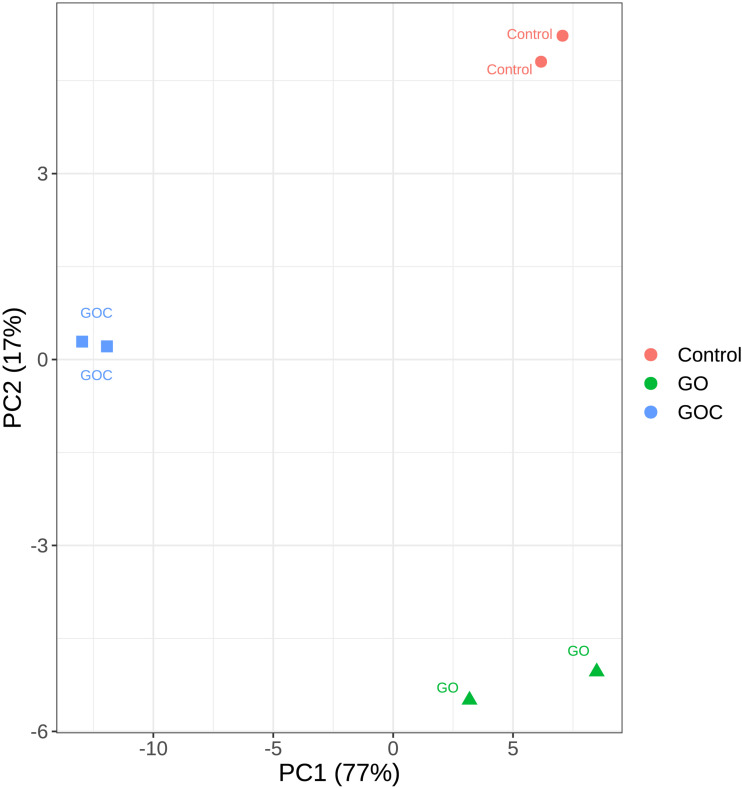
Principal Component Analysis plot of the transcriptomic response of *S. cerevisiae* to two different graphene oxide products at 160 mg L^–1^ (Control: non-exposed cells, GO: exposed to monolayer graphene, and GOC: exposed to graphene oxide nanocolloids). Only the top 500 genes with most variability were considered.

Principal Component Analysis (PCA) was performed to analyze the variability among the generated samples (Figure 2). In this analysis, only the top 500 genes with most variability were considered to reduced noise associated to biological variability. Similar results are obtained when all genes are considered, as shown in the PCA plot displayed in Supplementary Figure 1. Obvious clustering can be seen between samples corresponding to biological replicates. The dimensionality reduction can be considered adequate, as most of the variability (77%) is along the X axis, the first principal component (PC1). Samples corresponding to control (non-exposure) and exposure to 160 ml L^–1^ GO are closer together in terms of transcriptomic response than they are to samples exposed to the same concentration of GOC. This similarity becomes even more apparent if we consider that the difference between the control and the GO samples is mainly along the second PC, whereas differences between GOC exposed and control samples appear along both PC1 and PC2. This can be interpreted as GOC having more variability than GO against the control, as the PC1 axis carries much more variability than the PC2 one. GOC exposure shows a much higher transcriptomic response than GO exposure, even though in both cases the same compound is used.

**FIGURE 3:**
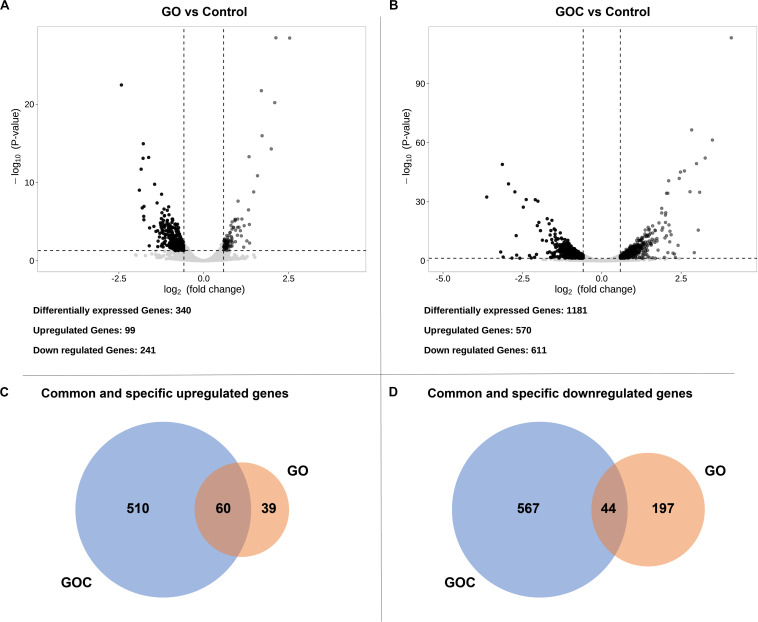
Volcano plots displaying the fold change (log2) of differentially expressed genes of GO vs. the control **(A)** and GOC vs. the control **(B)**, and Venn diagrams showing common and specific upregulated **(C)** and downregulated **(D)** genes between the different exposure conditions. The genes were considered significantly differentially expressed if they had a fold change higher than 1.5 (upregulated) or lower than 1/1.5 (downregulated), and an FDR lower than 0.05.

Afterward, both exposure conditions were studied individually to visualize the transcriptional impact of each compound on *S. cerevisiae*. Differentially expressed genes were defined (Supplementary Table 2) by a False Discovery Rate (FDR) lower than 0.05. To further reduce the number of false positives associated to the relatively low number of replicates, we have imposed a threshold on fold change, so that only differentially expressed genes higher or lower than 1.5 and 1/1.5, respectively (corresponding to ± 0.585 in log_2_), were considered biologically meaningful (Schurch et al., 2016). The obtained data was displayed in volcano plots (Figures 3A,B), where clear differences between both exposure conditions in number of genes differentially expressed could be observed. Yeast cells exposure to GOC induced more than three times more differentially expressed genes than GO exposure (1181 and 340 genes, respectively). Surprisingly, only a small part of the differentially expressed genes in both conditions was common (104, of which 60 were upregulated and 44 downregulated. This indicates a very specific transcriptional response of *S. cerevisiae* to each type of graphene oxide.

**FIGURE 4:**
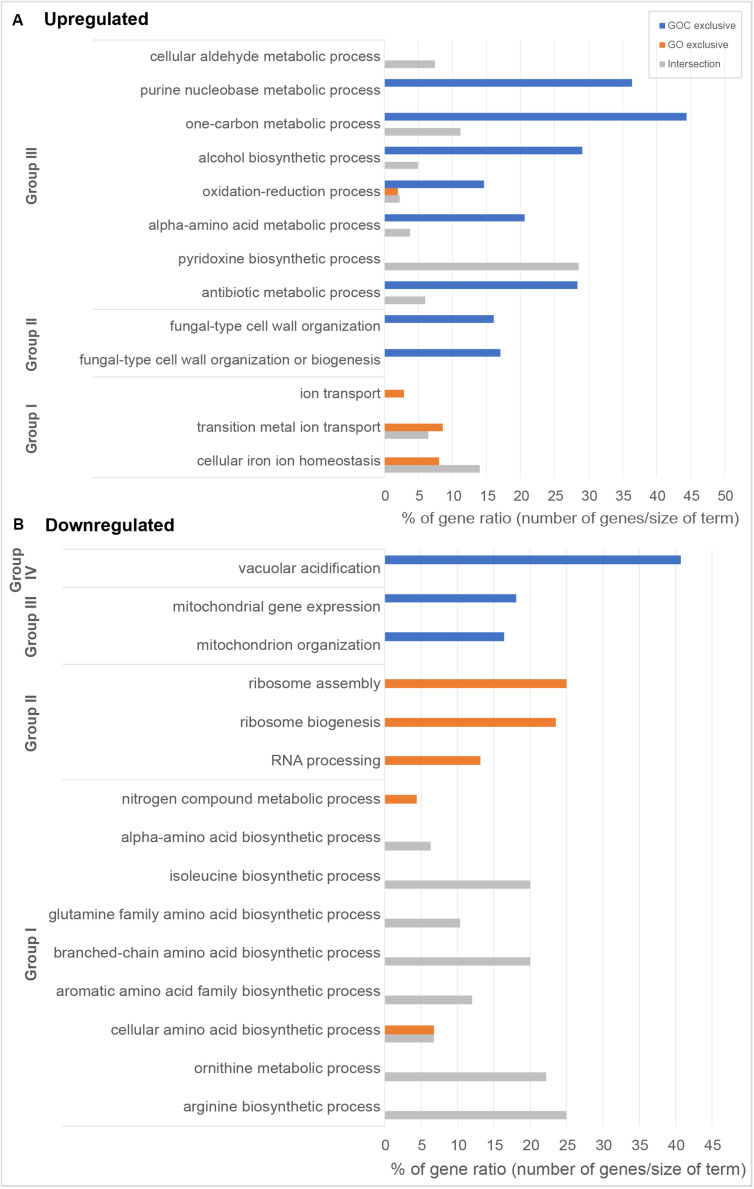
Overview of gene ontology enrichment analysis of *S. cerevisiae* **(A)** upregulated and **(B)** downregulated genes. Blue and orange bars indicate terms enriched among the genes exclusively differentially expressed upon GOC or GO exposures respectively, while gray is used for terms found among the genes commonly differentially expressed in both exposure conditions. Bar size indicates the % of genes in the whole genome annotated to the corresponding term that have been found in each of the exposures. Gene ontology entries have been grouped in terms broadly related to **(A)** Group I: metal bioavailability, Group II: cell wall structure, and Group III metabolism and in **(B)** Group I: amino acid metabolism, Group II: protein translation, Group III: mitochondria, and Group IV: vacuolar acidification. All selected terms have an FDR lower than 0.05.

Following the previous exploratory analysis, Gene Ontology enrichment and KEGG (Kyoto Encyclopedia of Genes and Genomes) pathway enrichment analysis of the differentially expressed genes were done. Both tests were performed separately for up and downregulated genes to study which biological functions were specifically altered upon yeast cells exposure to each nanomaterial, as well as to identify the common cellular response. Gene Ontology enrichment analyses were performed for each of the three ontologies: biological process (BP), molecular function (MF) and cellular component (CC). An overview of the results for the BP ontology is shown in Figure 4, whereas full results are available in Supplementary Table 3. This supplementary table also provides a full list of all the genes associated to the corresponding gene ontology terms and their functional annotation. The results of KEGG pathway enrichment analysis can be found in the Supplementary Table 4. Enrichments were considered significant whenever FDR <0.05.

Amongst the common upregulated genes (60, as shown in Figure 3C), there is a significant enrichment in genes associated to the Gene Ontoloy term “cellular iron ion homeostasis” (inside Group I, Figure 4A). The seven genes associated to this term commonly upregulated are involved in functions related to iron uptake at the cell surface, iron efflux from vacuole to cytosol and in metabolic adaptation to low iron conditions. YDR270W (*CCC2*) encodes a P-type copper-transporting ATPase necessary for the proper uptake of iron (Fu et al., 1995). The expression of YOL158C (*ENB1*), YHL047C (*ARN2*), and YOR384W (*FRE5*) is related to non-reductive and reductive iron transport systems (Martins et al., 1998; Heymann et al., 1999; Philpott et al., 2002). YLR136C (*TIS11*) and YLR205C (*HMX1*) are involved in mRNA and heme degradation, respectively mediating homeostatic changes, such as making heme iron available for metabolic needs, and reducing iron flux into respiratory complexes (Protchenko and Philpott, 2003; Puig et al., 2005). The expression of the mentioned genes is higher in iron starvation conditions, and it is controlled by the Aft1p and Aft2p regulators (Rutherford et al., 2003). The YLR136C (*CTH2*) gene, whose expression is also controlled by the Aft1/2 regulon and contributes to remodeling yeast metabolism by suppressing pathways employing many iron-containing enzymes, was also upregulated in the presence of GO and GOC (Matsuo et al., 2017). A role for *CTH2* in increasing resistance to ROS when this gene is overexpressed has been proposed (Matsuo et al., 2017). In case of YMR134W (*ERG29*), its function is related to ergosterol biosynthesis and has been tied to iron metabolism too (Moretti-Almeida et al., 2013). The common upregulation of YER037W (*PHM8*), which is involved in lysophosphatidic acid hydrolysis in response to phosphate starvation, suggests low availability of this nutrient too (Vardi et al., 2014). In addition, a common upregulation of transcriptional response to the presence of both nanomaterials was observed for genes annotated to the term “cellular aldehyde metabolic process” (Figure 4A, Group III): YGR256W (*GND2*), YMR095C (*SNO1*), YMR096W (*SNZ1*), and YNL117W (*MLS1*). *GND2* encodes a phosphogluconate dehydrogenase, which is induced in stress conditions and it could have a protective role against oxidative stress (Izawa et al., 1998). *SNO1* and *SNZ1* are members of a stationary phase-induced gene family, involved in pyridoxine (vitamin B6) biosynthesis, whose accumulation occurs as well in response to the limitation of specific nutrients and stress response to nucleotide imbalance (Padilla et al., 1998; Rodríguez-Navarro et al., 2002). Overexpression of these genes is also identified in the enrichment of the “Vitamin B6 metabolism” KEGG metabolic pathway (see Supplementary Table 4). Furthermore, the YDR019C (*GCV1*) and YMR189W (*GCV2*) genes, which code two of the three proteins involved in the glycine decarboxylase multienzyme complex, are induced by high levels of glycine or repressed in the presence of rich nutritional environments or high quality nitrogen sources (Sinclair et al., 1996; Piper et al., 2002). Alterations in nitrogen metabolism are also evident in the metabolic pathway enrichment analysis that shows dysregulation of metabolic pathways related to amino acid synthesis and degradation (Supplementary Table 4).

Regarding the 39 exclusively upregulated *S. cerevisiae* genes in the presence of monolayer graphene oxide (GO) (Figure 3C), additional genes related to metallic elements transport (“ion transport”; “transition metal ion transport,” shown in Figure 4A, Group I, and in Supplementary Table 3) were overexpressed: YMR058W (*FET3*), part of the high affinity iron uptake system in the cell wall (Askwith et al., 1994) and YKL220C (*FRE2*), a ferric and cupric reductase, which reduces siderophore-bound iron and oxidized copper prior to uptake by transporters, are involved in iron uptake (Elena and Despina, 1994); YHL040C (*ARN1*) and YEL065W (*ARN3*) are members of the ARN family transporters, which specifically recognize siderophore-iron chelates, and are induced in conditions of low iron (Heymann et al., 2006); YOR382W (*FIT2*) and YOR383C (*FIT3*) are cell wall glycosylphosphatidylinositol-anchored mannoproteins involved in the retention of siderophore-iron in the cell wall (Protchenko et al., 2001); YOR316C (*COT1*) is a vacuolar transporter that mediates zinc transport, but its expression levels are controlled by the iron regulon in yeast (Philpott and Protchenko, 2008); and YER053C (*PIC2*) belongs to the mitochondrial carrier family (MCF), involved in phosphate and copper transport (Vest et al., 2013).

Besides iron and other genes involved in metal homeostasis, the upregulation of YMR195W (*ICYI*), which is induced by amino acid starvation (Kleinschmidt et al., 2005), was observed too. It is also interesting to highlight the upregulation of two stress response genes: YMR175W (*SIP18*), regulated by osmotic stress, and YCR021C (*HSP30*), induced by heat shock and entry to the stationary phase (Panaretou and Piper, 1992; Miralles and Serrano, 1995; see Supplementary Table 2). Few genes related to one carbon metabolism [YER081W (*SER3*) and YCL064C (*CHA1*)] and glycogen metabolism [YMR105C (*PGM2*), YIL050W (*PCL7*), and YJL137C (*GLG2*)] were found upregulated too in the GO condition.

Amongst the high number of exclusive upregulated genes (510, as shown in Figure 3C) in yeast cells exposed to graphene nanocolloids (GOC), 13 of those were specifically related to the ergosterol biosynthetic process (see Supplementary Table 3). Genes from the mentioned pathway were found to be overexpressed during iron starvation conditions in a previous study (Puig et al., 2005). Other authors studying the metabolic response to iron deficiency in *S. cerevisiae* only observed small changes in the transcript levels of *EFG* genes, but specific alterations in the ergosterol and sphingolipid biosynthetic pathways steps involving heme and diiron enzymes were found (Shakoury-Elizeh et al., 2010). The presence of GOC activated additional specific and general responses related to the low availability of other nutrients, such as zinc, phosphate, nitrogen and pyrimidine (YML123C, YBL042C, YJL056C, YPR035W, YOR030W, YLR014C, YNR002C, YKR042W, YIL101C, YGL180W).

The upregulation of many genes involved in the maintenance of cell wall integrity (see Figure 4A, Group II, and Supplementary Table 3), some of them induced in response to stress, such as YDR077W (*SED1*), YGR189C (*CRH1*), YLR194C (*NCW2*), YPR026W (*ATH1*), and YJL159W (*HSP150*), was also observed. Aggregation, morphological alterations, gemmation disturbance and in some cases cellular damage has been reported upon exposure of *S. cerevisiae* cells to graphene oxide (Zhu et al., 2017). Similarly, alterations at cell wall integrity at molecular level were also recently observed in the filamentous fungus *Fusarium graminearum* in the presence of different graphene oxide concentrations (Wang et al., 2019). Previous reports have highlighted the ability of the nanomaterial to intertwine with unicellular microbial systems (bacteria and fungal spores), probably causing structural damages of cell wall and plasma membranes (Chen et al., 2014).

Several genes described to have a role upon oxidative stress showed to be upregulated in the presence of GOC: YDL010W (*GRX6*), YGR154C (*GTO1*), YPL061W (*ALD6*), YKL086W (*SRX1*), YGR023W (*MTL1*), and YLR380W (*CSR1*) (see Supplementary Table 2 for exact values of fold change). Many studies have identified the production of reactive oxygen species as a common mechanism of carbon derived nanomaterials (graphene derivatives, carbon nanotubes, etc.) to induce cell toxicity in microbial and unicellular systems (Chen et al., 2019; Madannejad et al., 2019). Therefore, similar responses at transcriptional level have been described in research works studying the interaction between microorganisms and carbon derived nanomaterials (Zhu et al., 2016; Chen et al., 2017; Yu et al., 2017; Suarez-Diez et al., 2020). An additional detailed inspection of the results also showed the overexpression of a significant number of genes involved in alpha-amino acid biosynthetic process (23), antibiotic metabolic process (19), alcohol biosynthetic process (18), one-carbon metabolic process (8), and purine nucleobase biosynthetic process (8), which suggest that GOC induced severe changes in the physiological state of the yeast.

In relation to the significantly downregulated genes found in *S. cerevisiae* cells exposed to GO and GOC, 44 of them where common to both conditions (Figure 3D), most of them with functions related to biosynthetic and metabolic processes related to amino acids biosynthesis, some of them shown in Figure 4B, Group I, such as the “isoleucine biosynthetic process,” “arginine biosynthetic process,” “aromatic amino acid family biosynthetic process,” and the “ornithine metabolic process,” which could be associated to deficiencies in specific iron-dependent enzymes involved in amino acid biosynthesis or the aforementioned low availability of nitrogen (Shakoury-Elizeh et al., 2010). For instance, the synthesis of branched-chain amino acids is subjected to iron availability due to the Fe/S proteins specifically involved in the pathway (Ihrig et al., 2010). One of them, the dihydroxyacid dehydratase YJR016C (*ILV3*), which catalyzes the third step in the common pathway leading to biosynthesis of leucine, isoleucine and valine, was downregulated upon exposure to both nanomaterials.

The transcriptional changes in genes associated to low nutrient availability in the presence of GO and GOC could be related to the capacity of these nanomaterials to adsorb biomolecules and ions, lowering their availability for biological systems. On one hand, the high protein adsorption capacity of GO and GOC has been recently described, which could have an impact on nitrogen availability in yeast cells (Antón-Millán et al., 2018; Domi et al., 2019). Also, iron sequestration by graphene oxide in yeast growth medium was previously described in a similar study were *S. cerevisiae* cells were exposed to a non-commercial sample of the nanomaterial (Yu et al., 2017). The ability of graphene oxide to adsorb iron was shown to be significantly higher than that of reduced graphene oxide, probably due to the difference in oxygen containing groups on the surface of both nanomaterial types, which makes the former nanomaterial type more reactive. The same observations were done by Antón-Millán et al. (2018) and Domi et al. (2019), when comparing the protein adsorption capacity of graphene oxide with that of lower oxygen containing carbon derived nanomaterials, such as polycarboxylate functionalized graphene nanoplatelets and reduced graphene oxide, respectively. Nevertheless, Suarez-Diez et al. (2020) also observed transcriptional evidence of metal ions deficiency (including iron) when yeast cells were exposed for 2 h to high concentrations of polycarboxylate functionalized graphene nanoplatelets (800 mg L^–1^), but not when their concentration was five times lower. The present study confirms previous observations done by Yu et al. (2017) and Suarez-Diez et al. (2020) at transcriptomics level, suggesting that nutrient sequestration by graphene derived nanoparticles could provoke potential adverse effects on the physiological state of microbial systems.

As previously described for the upregulated genes in both exposure conditions, most of downregulated genes were specific for GO or GOC (Figure 3D). In case of the GO condition, 197 genes were exclusively downregulated, most of them with functions related to the rRNA processing and ribosomal assembly (Figure 4B, Group II, and Supplementary Table 3). Ribosomal protein (RP) genes, coding for structural components of cytoplasmic ribosomes, and ribosome biogenesis (Ribi) genes, are among the largest yeast regulons and are subjected to strict transcriptional regulation through various nutrient and stress signaling pathways (Bosio et al., 2017). In fact, different toxicology studies in *S. cerevisiae* have reported similar results in response to different stress inducing conditions (Yu et al., 2010; Bereketoglu et al., 2017; Soontorngun, 2017). In particular, iron starvation has been recently shown to be responsible for the decrease in the transcription rates of RP and RiBi genes, through the inhibition of one of the major nutrient-sensing kinase pathways, such as the target of rapamycin complex 1 (TORC1) (Romero et al., 2019). However, since TORC1 activity is also regulated in response to different nutrient limiting conditions (carbon, nitrogen, phosphate) and other harmful stressors (high salt, redox stress, a shift to a higher temperature, or caffeine) (Loewith and Hall, 2011), the potential inhibition of this complex when yeast is exposed to graphene oxide nanomaterials could be due to more environmental factors in addition to iron limiting conditions. In this regard, it is interesting to remark that yeast cells exposed for 2 h to 160 mg L^–1^ of polycarboxylate functionalized graphene nanoplatelets did not show transcriptional evidence of iron starvation or other nutritional stresses, but similarly to what was observed in the present study, a high number of RP and Ribi genes were found to be downregulated too (Suarez-Diez et al., 2020).

In relation to GOC, a higher number of genes (567) were exclusively downregulated in response to this nanomaterial (Figure 3D). Many of them were linked to mitochondria and mitochondrial activity, as indicated in Figure 4B, Group III, and in Supplementary Table 3. For instance, mitochondrial translation genes (36), mitochondrial transport genes (17) and respiratory complex assembly genes (12) were significantly downregulated. Many toxicity studies have reported the ability of nanomaterials from different origin to damage mitochondrial structure and function (Wu et al., 2020). In particular, SWCNTs, MWCNTs and graphene oxide have been reported to reduce mitochondrial membrane potential in yeast (Zhu et al., 2016, 2017, 2018), as in many other eukaryotic cellular models (Wu et al., 2020). The reduction of mitochondrial activity has been related to decreased ROS production associated to mitochondrial respiratory reactions, through a mechanism mediated by *CTH2*, which is activated in response to iron-scarcity conditions (Matsuo et al., 2017). Additionally, the function of a high number of genes was associated to chromosome segregation (Supplementary Table 3). This mechanism is blocked in *S. cerevisiae* when DNA replication is challenged. For instance, several genes controlled by the CLB2 cluster (e.g., YGR108W, YDR146C, YGL116W, YIL158W, YNL058C, etc.) were downregulated, a process that has been associated as well to DNA replication stress induced by genotoxic conditions (Palou et al., 2015). Interestingly, genes related to the process of vacuolar acidification (11) showed a lower expression level too (Figure 4B, Group IV). Several genes of the proton pump vacuolar ATPase (V-ATPase) complex, which controls intracellular and extracellular pH, were found to be downregulated (YGR020C, YBR127C, YEL051W, YEL027W, YHR026W, YHR039C-A, YCL005W-A) as well as some connected to its function (YKL119C, YHR060W). V-ATPases acidify endosomes and lysosomes by pumping protons from the cytoplasm to their lumen, promoting iron mobilization and utilization (Diab and Kane, 2013). If regulated incorrectly, iron may react with H_2_O_2_, generating hydroxyl radicals and provoking cellular damage. A lower activity of this complex generates an iron deprivation signal, inducing the iron regulon. Also, an acidic cytosolic environment could promote iron bioavailability (Diab and Kane, 2013).

The results obtained in the present study show common and distinct cellular responses to two very similar commercial graphene oxide products, indicating that small disparities in manufacturing processes can result in a specific and divergent responses to these nanomaterials from biological systems. Small undetected distinct morphological features or observed differences in elemental composition might influence the nanomaterials reactivity, allowing them to elicit common and specific transcriptional responses in yeast. Both nanomaterials induced common and specific responses associated to iron scarcity and other stress factors. Significant common and specific changes in genes linked to homeostasis and ribosomal indicate major changes in the physiological state of yeast cells in the presence of these nanomaterials. The reported results contribute to understand the physiological response of fungal cells to the presence of graphene oxide, highlighting the relevance of determining the biological response of potentially exposed organisms to specific commercial nanomaterials.

## Data Availability Statement

The transcriptomics datasets generated and analyzed for this study can be found in the European Nucleotide Archive under accession number PRJEB34525.

## Author Contributions

JT-R conceived and designed the work. JT-R, MS-D, and FL-T performed the experiments, analyzed and interpreted the data, and critically revised the manuscript for intellectual content. JT-R and FL-T drafted the manuscript. All authors contributed to the article and approved the submitted version.

## Conflict of Interest

The authors declare that the research was conducted in the absence of any commercial or financial relationships that could be construed as a potential conflict of interest.
