# Supplementary material for: Commonalities and Differences in the Transcriptional Response of the Model Fungus Saccharomyces cerevisiae to Different Commercial Graphene Oxide Materials
Source: Front Microbiol. 2020 Aug 11;11:1943. doi: 10.3389/fmicb.2020.01943 (PMC7431627; doi:10.3389/fmicb.2020.01943)
Supplement: Supplementary file 1 [file Data_Sheet_1.zip › Figure S1.DOCX]

Supplementary Material

Commonalities and differences in the transcriptional response of the model fungus Saccharomyces cerevisiae to different commercial graphene oxide materials

Felix Laguna-Teno^1^, Maria Suarez-Diez^2^, Juan Antonio Tamayo-Ramos^1*^

^1^ International Research Centre in Critical Raw Materials-ICCRAM, University of Burgos, Plaza Misael Banuelos s/n, 09001 Burgos, Spain.

^2^ Laboratory of Systems and Synthetic Biology, Wageningen University & Research, Stippeneg 4 6708WE Wageningen, the Netherlands.

*** Correspondence:**Juan Antonio Tamayo-Ramos
ja.tamayoramos@gmail.com


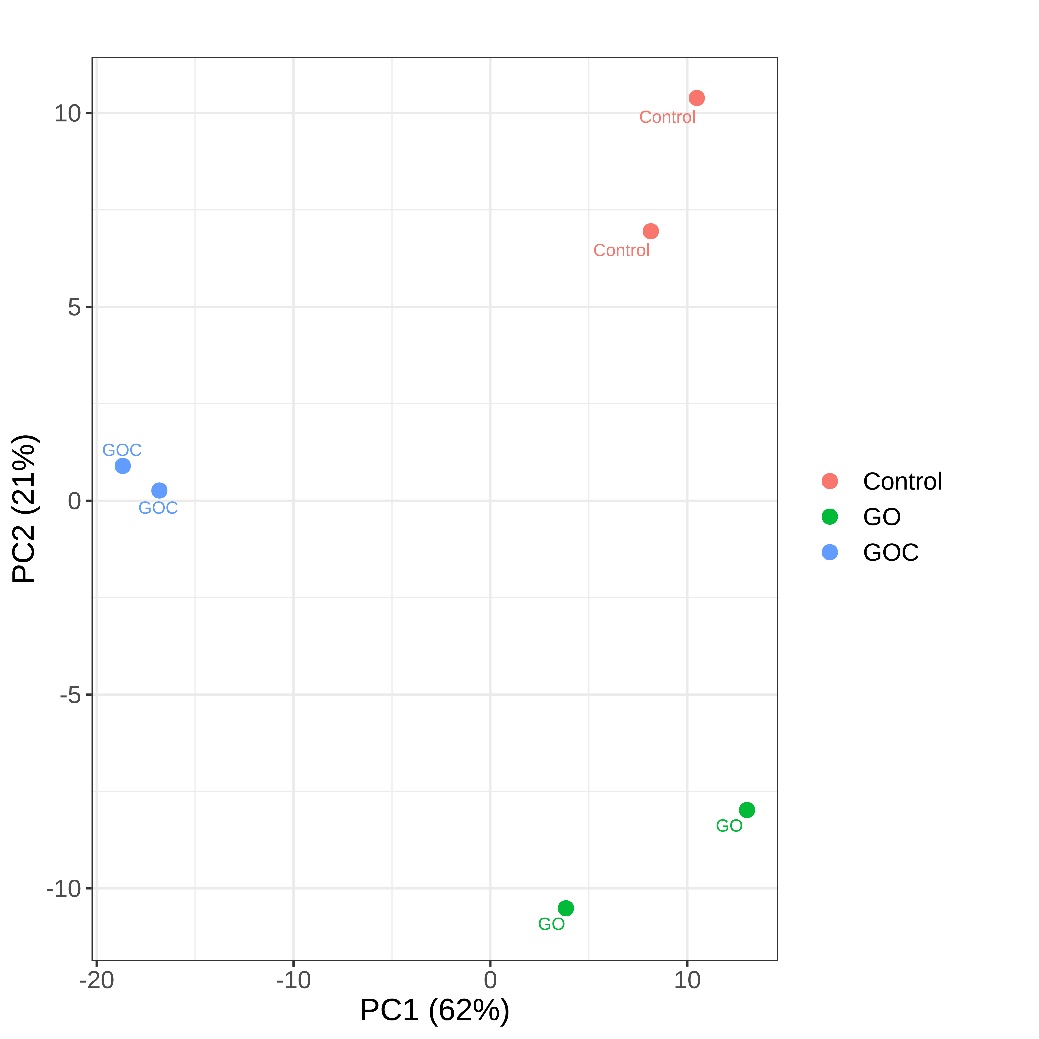


**Supplementary Figure 1. Principal Component Analysis plot.**

PCA plot of the transcriptomic response of *S. cerevisiae* to two different graphene oxide products at 160 mg L^-1^ (Control: non non exposed cells, GO: exposed to monolayer graphene oxide, and GOC: exposed to graphene oxide nanocolloids). All genes with significant expression changes are considered.
